# A Nomogram for Preoperative Estimation of Microvascular Invasion Risk in Hepatocellular Carcinoma: Single-Center Analyses With Internal Validation

**DOI:** 10.3389/fonc.2021.616976

**Published:** 2021-03-04

**Authors:** Jiarui Yang, Shuguang Zhu, Juanjuan Yong, Long Xia, Xiangjun Qian, Jiawei Yang, Xueqiao Hu, Yuxuan Li, Chusi Wang, Wenguang Peng, Lei Zhang, Meihai Deng, Weidong Pan

**Affiliations:** ^1^Department of Biliary-Pancreatic Surgery, The Third Affiliated Hospital of Sun Yat-sen University, Guangzhou, China; ^2^Department of Hepatic Surgery and Liver Transplantation Center, The Third Affiliated Hospital of Sun Yat-Sen University, Guangzhou, China; ^3^Department of Pathology, Sun Yat-Sen Memorial Hospital, Sun Yat-Sen University, Guangzhou, China; ^4^Department of Hepatobiliary Surgery, The Third Affiliated Hospital of Sun Yat-sen University, Guangzhou, China

**Keywords:** hepatocellular carcinoma, microvascular invasion, prediction model, nomogram, internal validation

## Abstract

**Background:**

Microvascular invasion (MVI) is highly associated with poor prognosis in patients with liver cancer. Predicting MVI before surgery is helpful for surgeons to better make surgical plan. In this study, we aim at establishing a nomogram to preoperatively predict the occurrence of microvascular invasion in liver cancer.

**Method:**

A total of 405 patients with postoperative pathological reports who underwent curative hepatocellular carcinoma resection in the Third Affiliated Hospital of Sun Yat-sen University from 2013 to 2015 were collected in this study. Among these patients, 290 were randomly assigned to the development group while others were assigned to the validation group. The MVI predictive factors were selected by Lasso regression analysis. Nomogram was established to preoperatively predict the MVI risk in HCC based on these predictive factors. The discrimination, calibration, and effectiveness of nomogram were evaluated by internal validation.

**Results:**

Lasso regression analysis revealed that discomfort of right upper abdomen, vascular invasion, lymph node metastases, unclear tumor boundary, tumor necrosis, tumor size, higher alkaline phosphatase were predictive MVI factors in HCC. The nomogram was established with the value of AUROC 0.757 (0.716–0.809) and 0.768 (0.703–0.814) in the development and the validation groups. Well-fitted calibration was in both development and validation groups. Decision curve analysis confirmed that the predictive model provided more benefit than treat all or none patients. The predictive model demonstrated sensitivity of 58.7%, specificity of 80.7% at the cut-off value of 0.312.

**Conclusion:**

Nomogram was established for predicting preoperative risk of MVI in HCC. Better treatment plans can be formulated according to the predicted results.

## Introduction

Hepatocellular carcinoma (HCC) is the sixth most common cancer and ranks as the third tumor-related death in the world (1). Although HCC can potentially be cured through resection or transplantation at early stage, most patients lost opportunities for curative surgical treatment because of liver dysfunction or disease extension (2). HCC recurrence occurs in nearly 70% of patients within 5 years (3).

Vascular invasion is one of the most crucial factors for poor prognosis after operation for HCC (4). It can be divided into macrovascular invasion (vascular invasion) and microvascular invasion (MVI). The invasion of cancer cell nest in the endothelial vascular lumen is defined as MVI (5). MVI is difficult to identify by preoperative imagine, and it can only be affirmed by postoperative pathology with little value in preoperative treatment management.

Lots of efforts have been taken to explore the relationships between preoperative parameters and MVI. Tumor diameter has been reported to be predictors for MVI in HCC patients (6, 7). However, the cut-off values of tumor diameter are inconsistent and they are just simply divided by equidistance. The use of serum or tumor biomarkers (such as AFP) to estimate MVI risk has also been reported (7, 8). Unfortunately, these serum markers can also elevate in HCC without MVI. Although some radiomic signatures have been shown to predict MVI risk, they were difficult to apply in clinical work (9). Therefore, we retrospectively analyzed clinical, imagine features and the feeling of patients with HCC to explore predictive factors of MVI in this study. We also developed a preoperative prediction model for MVI and validated it by internal validation.

## Materials and Methods

### Patients

Four hundred five consecutive HCC patients who received liver resection were prospectively collected at the Third Affiliated Hospital of Sun Yat-sen University, between January 1, 2013, and December 31, 2015. The inclusion criteria were (1) undergone R0 tumor resection as defined in a previous report (10), (2) MR enhanced scan of the liver was performed within 1 month before resection, (3) Child-Pugh A or B. The exclusion criteria were: (1) HCC with satellite lesions, portal vein tumor thrombus, or extrahepatic metastasis, (2) patients with previous anticancer treatment, (3) patients with other malignancies prior to surgery, (4) incomplete clinical data. Two hundred ninety eligible patients were included into the development group for developing the nomogram; one hundred fifteen patients were entered into the validation group. The assignment of development and validation groups was based on random number generated by package “caret” of R software and the random number generation code was followed by the instruction of package “caret”. The two groups were randomly assigned by a ratio of 7:3. The random number was 20191218.

### Laboratory Test and Pathological Characteristics

Basic information of admission included the discomfort of right upper abdomen, routine preoperative laboratory examination, preoperative liver function tests, HBV DNA load, and *α*-fetoprotein (AFP) levels were collected from medical records, ultrasonography, MRI, and CT of the abdomen. All surgical specimens were routinely examined histopathologically. Each specimen was cut continuously at the maximum diameter, fixed within 30 min after removal, and then seven-point baseline sampling was performed (11). Cancer cell nest in the endothelial vascular lumen was defined as MVI, including intra-tumoral and extra-tumoral MVI.

### Statistical Analysis

T-test was used to analyze parametric data of component numerical variables, and Mann–Whitney rank sum test was used to analyze non-parametric data. Fisher’s exact test or chi square test was used to compare the categorical variables. The sensitivity and specificity were estimated by the ROC numerical integration. The optimum subsection of each numerical variable is obtained by the ROC and the optimal scale regression analysis. P less than 0.05 represented statistical significance. The above analyses were performed by SPSS 23.0 software (IBM corporation, 2015, USA). Application of lasso regression analysis was used to select the best predictors of liver cancer in patients with MVI and the non-zero coefficient factor should be selected (12). All possible predictive factors were performed to construct a simplified model for preoperative prediction of MVI. A simplified nomogram is shown based on the results of LASSO regression analyses. The discrimination performance of the nomogram was quantified by the Harrell’s concordance index (C-index) and the discrimination was relatively good when the value of C-index was higher than 0.75 (13). Calibration curves were conducted to estimate the predictive efficacy of the predictive model (14). Decision curve was carried out to calculate the net benefits of individuals in different threshold probabilities and confirm clinical efficacy of the predictive model (15). The net benefit was calculated by subtracting all false positive patients’ proportion from the true positive patients’ (16). And it was a decision analysis method that put benefits and harms on the same scale (17). The above statistical methods were carried out with R software version 3.6.1 (http://www.r-project.org) with R software packages “Hmisc”, “rms”, “ROCR”, “rmda”, “caret”, “glmnet” and “foreign”.

## Results

### Patients Characteristics

The baseline data of 405 patients were listed in **Table 1**; 290 and 115 patients were divided into the development and validation groups, respectively. There was no significant difference in baseline clinicopathological data between the development and validation groups. MVI was found in 123 (42.4%) and 45 (39.1%) patients in the two groups, respectively.

**Table 1:** Patients Characteristics.

Variable		Group, No. (%)		P value
		Development	Validation	
		(n = 290)	(n = 115)	
Age (years)		58 ± 17	50 ± 19	0.066
Sex	Male	252 (86.9)	99 (86.1)	0.829
	Female	38 (13.1)	16 (13.9)	
Hypertension	Presence	32 (11.0)	8 (7.0)	0.215
	Absence	258 (89)	107 (93)	
HBsAg	Positive	259 (89.3)	104 (90.4)	0.738
	Negative	31 (10.7)	11 (9.6)	
Discomfort of upper right abdomen	Presence	97 (33.4)	46 (40.0)	0.214
	Absence	193 (66.6)	69 (60.0)	
Antivirus^a^	Presence	71 (24.5)	25 (21.7)	0.558
	Absence	219 (75.5)	90 (78.3)	
Smoke	Presence	88 (30.3)	41 (35.7)	0.301
	Absence	202 (69.7)	74 (64.3)	
Drink	Presence	42 (14.5)	10 (8.7)	0.116
	Absence	248 (85.5)	105 (91.3)	
Liver cirrhosis	Presence	206 (71.0)	80 (69.6)	0.77
	Absence	84 (29.0)	35 (30.4)	
Child-Pugh	A	275 (94.8)	111 (96.5)	0.467
	B	15 (5.2)	4 (3.5)	
Tumor number	Solitary	237 (81.7)	99 (86.1)	0.292
	Multiple	53 (18.3)	16 (13.9)	
Tumor location	Right lobe	192 (66.2)	83 (72.2)	0.246
	Left lobe	98 (33.8)	32 (27.8)	
Vascular invasion	Presence	80 (27.6)	39 (33.9)	0.208
	Absence	210 (72.4)	76 (66.1)	
Lymphatic metastasis	Presence	31 (10.7)	11 (9.6)	0.649
	Absence	259 (89.3)	104 (90.4)	
Tumor boundary^b^	Smooth	173 (59.7)	60 (52.2)	0.738
	Not smooth	117 (40.3)	55 (47.8)	
Tumor necrosis	Presence	69 (23.8)	26 (22.6)	0.170
	Absence	221 (76.2)	89 (77.4)	
HBVDNA, IU/ml	<100	113 (39.1)	39 (34.5)	0.800
	>100	176 (60.9)	74 (65.5)	
Microvascular invasion	Presence	123 (42.4)	45 (39.1)	0.394
	Absence	167 (57.6)	70 (60.9)	
Tumor diameter (mm)		37 ± 27	35 ± 23	0.545
lnα-Fetoprotein (ng/ml)		4.39 ± 4.76	4.08 ± 5.13	
Carcinoma embryonic antigen (μg/L)		1.88 ± 2.05	0.80 ± 4.30	
FER (μg/L)		296.10 ± 297.10	275.30 ± 121.50	0.854
CA199 (U/ml)		7.32 ± 15.21	3.28 ± 15.07	0.966
CA125 (U/ml)		11.62 ± 8.54	10.71 ± 4.12	0.330
CA153 (U/ml)		10.31 ± 7.73	8.93 ± 3.61	0.204
White blood cells (×10^9^/L)		5.56 ± 2.09	4.82 ± 1.99	0.672
Red blood cell (×10^12^/L)		4.53 ± 0.98	4.67 ± 0.57	0.769
Hemoglobin (g/L)		131.63 ± 22.11	145.36 ± 13.81	0.062
Platelets (×10^9^/L)		155.01 ± 106.23	177.73 ± 22.78	0.037
NEUT# (×10^9^/L)		3.03 ± 1.71	2.96 ± 0.65	0.127
LYMPH# (×10^9^/L)		1.74 ± 0.54	1.77 ± 0.57	0.830
MONO# (×10^9^/L)		0.43 ± 0.26	0.41 ± 0.09	0.405
Glutamic oxalacetic transaminase (U/L)		35.13 ± 23.02	30.32 ± 34.02	0.605
Alanine aminotransferase (U/L)		30.00 ± 34.00	34.00 ± 12.00	0.049
Albumin (g/L)		38.57 ± 3.81	40.24 ± 3.26	0.068
Total bilirubin (μmol/L)		14.50 ± 9.65	8.80 ± 10.40	0.194
Direct bilirubin (μmol/L)		5.00 ± 3.00	3.00 ± 3.70	0.533
Glutamyltranspeptidase (U/L)		57.00 ± 62.00	32.00 ± 31.00	0.087
Alkaline phosphatase (U/L)		84.00 ± 36.50	86.00 ± 19.00	0.097
Total bile acid (μmol/L)		7.70 ± 10.85	5.60 ± 7.10	0.122
Urea nitrogen (mmol/L)		4.91 ± 2.20	5.99 ± 2.32	0.818
Creatinine (μmol/L)		78.80 ± 17.00	83.00 ± 19.3	0.536
Cholinesterase (U/L)		6,285.29 ± 1,700.60	7,459.73 ± 2578.0	0.149
Pre-albumin (mg/L)		165.45 ± 51.3	208.91 ± 39.40	0.162
Alpha-l-fucosidase (U/L)		35.13 ± 17.21	33.04 ± 9.24	0.436
Prothrombin time (s)		14.03 ± 1.30	13.60 ± 0.900	0.298
APTT (sec)		38.60 ± 5.10	38.20 ± 4.00	0.838

APTT, activated partial thromboplastin time.

^a^Antiviral therapy was given before surgery.

^b^Tumor boundary on imaging was categorized as (1) smooth, presenting as a nodular-shaped tumor on all axial, coronary, and sagittal imaging or (2) not smooth, presenting as single nodule with no clear boundary.

### Factor Selection

**Figure 1:**
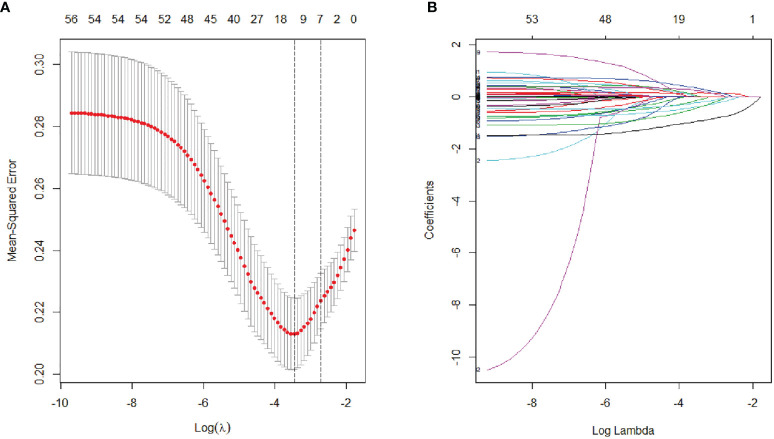
Lasso binary logistic regression analysis to select predictors of MVI. **(A)** The optimal parameter (*λ*) of Lasso is selected by the minimum criterion for five times cross validation. The relationship between partial likelihood deviation (binomial deviation) and logarithm (*λ*). By executing the minimum criterion and the 1 SE (1-SE criterion) of the minimum criterion, a dashed line was displayed at the optimal value. **(B)** The distribution of lasso coefficient of fifty-six factors. The coefficient distribution is calculated according to the logarithmic (*λ*) sequence. Vertical lines were shown at the values selected using cross validation, where the best *λ* produced seven factors with non-zero coefficients.

The variables used in LASSO regression analyses were collected from the data obtained preoperatively. Diameter, number, boundary and necrosis of tumor were extracted by preoperative imaging. LASSO regression analyses results were presented in **Figure 1**. Seven factors were found to be related with the MVI. These factors included discomfort of right upper abdomen, vascular invasion, lymphatic metastasis, tumor boundary, tumor diameter, tumor necrosis, and alkaline phosphatase (ALP). Discomfort of right upper abdomen had already excluded the discomfort caused by biliary tract disease or stomach illness. Vascular invasion and lymphatic invasion were the focus which could be observed directly by MRI. Tumor diameter was divided into four grades by optimal scale regression analysis (10–35, 35–65, 65–120, 120–220 mm).

### Constructing MVI Preoperative Prediction Nomogram

**Figure 2:**
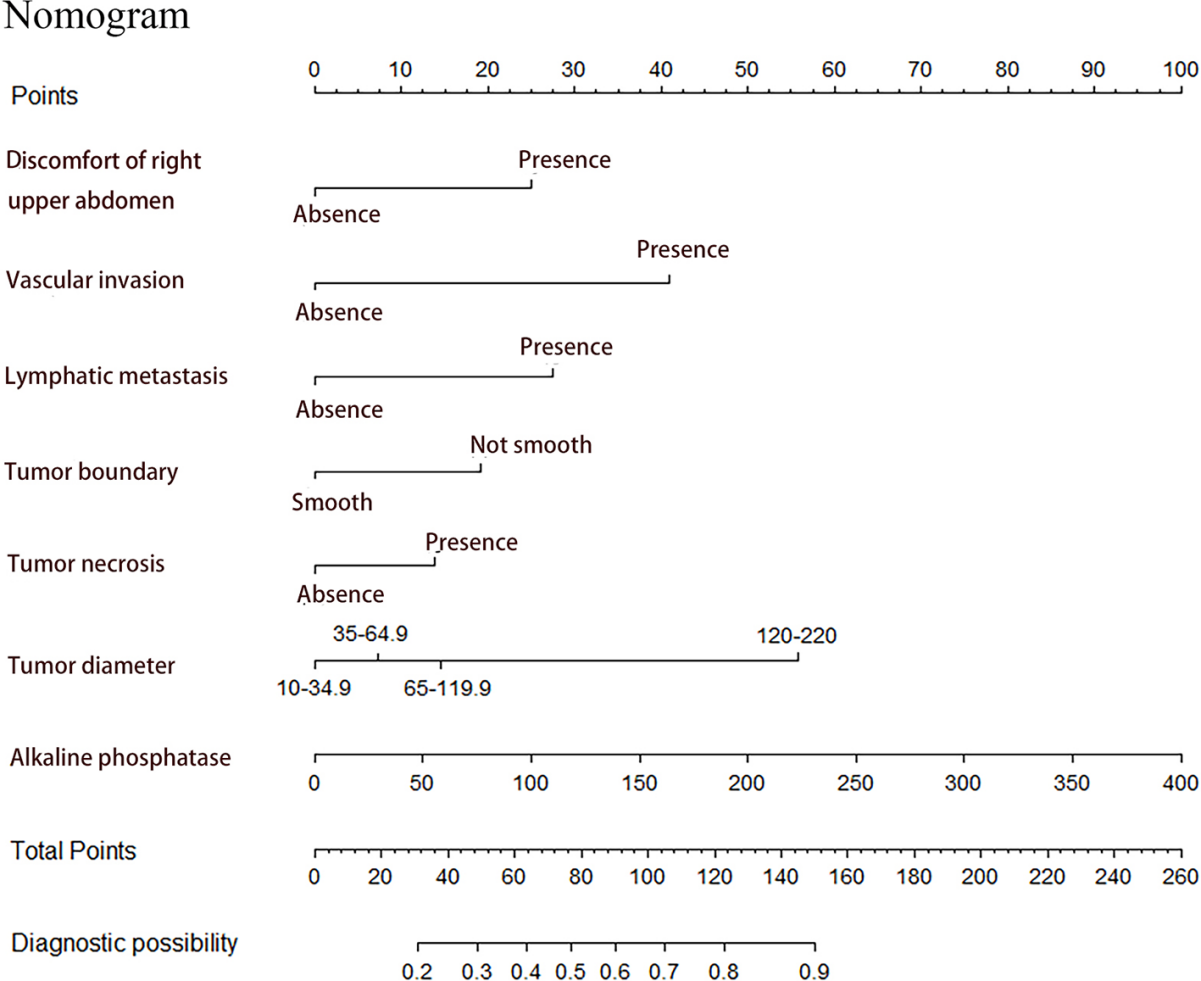
Prediction of MVI in patients with hepatocellular carcinoma by nomogram. In order to get every factor’s position on the corresponding axis, lines were drawn on the point axis to represent the number of points. Added all points, find the position of the total score to determine the MVI probability of that line in the nomogram.

**Figure 3:**
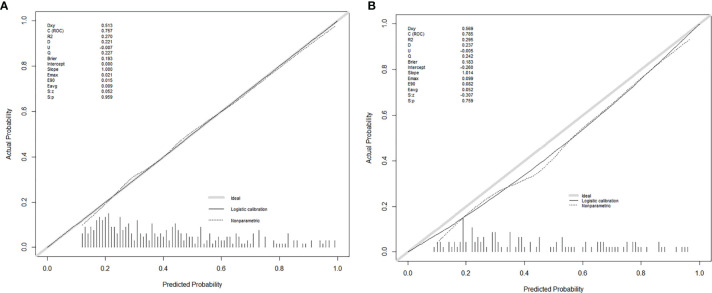
The calibration curves for predicting MVI in the development group **(A)** and the validation group **(B)**, respectively. Nomogram-predicted MVI was plotted on the X-axis, and the actual MVI occurrence was plotted on the Y-axis. A plot along the 45° line would indicate a perfect calibration model in which the predicted MVI is identical to the actual MVI. The distribution of the predicted probabilities of MVI occurrence was shown at the top of the graphs.

**Figure 4:**
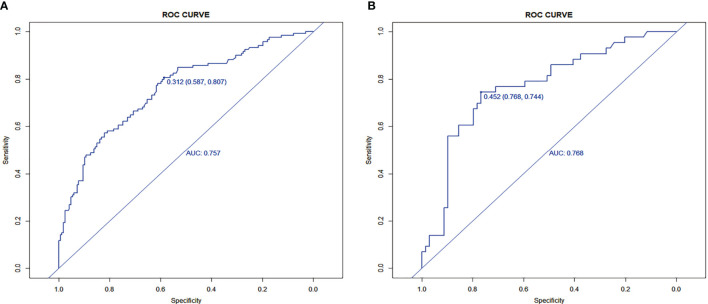
The ROC curves in **(A)** the development group (0.757) and **(B)** the validation group (0.768).

Seven factors chosen by LASSO regression analyses were used to construct a MVI predictive nomogram (**Figure 2**). The calibration curves showed a good agreement (**Figure 3**) in both development and validation groups. The C-index was 0.757 (0.723–0.792) in the development group and 0.768 (0.718–0.803) in the validation group. The results indicated an acceptable discrimination capability. The ROC curve in development and validation groups were showed in **Figure 4**. The AUC values of the nomogram were 0.757 and 0.768 in the development and validation groups, respectively.

**Figure 5:**
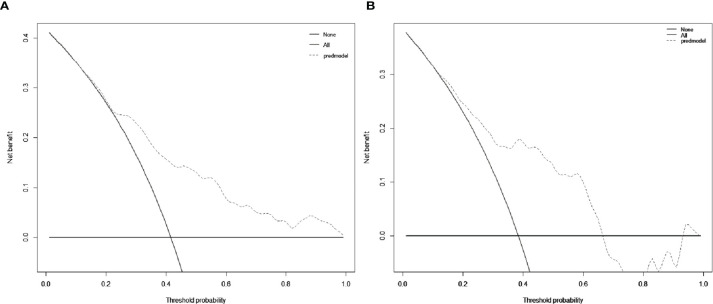
Decision curve analysis (DCA) for the nomogram in the development group **(A)** and the validation group **(B)**, respectively. The black solid lines hypothesized that all patients were MVI positive or negative, respectively. The dotted-line represented the net benefit of the nomogram at different threshold probabilities.

The decision curve was exhibited in **Figure 5**. When the cut-off value was 0.312, the net benefit was 20.5 and 18.0 in the development and validation groups, respectively.

### Risk of MVI Based on the Nomogram

The optimal cut-off value of the nomogram was 0.312. The sensitivity and specificity when used in differentiating the presence from absence of MVI were 58.7 and 80.7% in the development group, and 76.8 and 74.4% in the validation group, respectively.

## Discussion

It was difficult to predict MVI preoperatively because MVI lacked specific clinical features and imaging characteristic. We conducted a model to preoperatively predict MVI in HCC patients in this study. Furthermore, we performed an internal validation to evaluate the quality of the nomogram. The nomogram incorporated discomfort of right upper abdomen, vascular invasion, lymphatic metastasis, tumor boundary, tumor diameter, tumor necrosis and alkaline phosphatase (ALP), which exhibited a good accuracy for predicting MVI. All factors used in the nomogram to predict MVI were easy-acquired, non-invasive for patients.

Previous studies (6, 7) used tumor boundary as well as diameter to predict MVI, but further clinical validation was required. In addition, we represented the first study to report the discomfort of the right upper abdomen for preoperative prediction of MVI. After excluding other reasons for discomfort of the right upper abdomen, such as biliary tract, gallbladder disease, and stomach disease, our results indicated that discomfort of the right upper abdomen related to MVI. Discomfort of the right upper abdomen suggested the injury of liver. In our study the proportion of discomfort of the right upper abdomen was 59 and 32% in HCC patients with or without MVI, respectively.

The gold standard that diagnosed MVI is histopathological examination after surgical resection and MVI couldn’t be observed by imaging (11). Therefore, if we observed vascular invasion in HCC by imaging examination, we still couldn’t diagnosis MVI. In our study, 47% of patients who showed MVI by pathologic examination after surgery had vascular invasion by gross examination before surgery. In contrast, only 17% of patients who did not show MVI on pathologic examination after surgery had vascular invasion by gross examination before surgery. 66% of patients who were found with vascular invasion were found to have MVI by histopathological examination after surgery. Although there was no report on the association between vascular invasion and MVI, Alberto et al. (18). reported that intravascular free-floating tumor cluster formed by vascular invasion may act like MVI. It supported our findings that vascular invasion was an important factor associated with MVI.

According to histology, MVI-positive tumor had an aggressive tendency to invade the tumor capsule and lymphatic nodules, which led to irregular tumor boundary and lymphatic metastasis (19, 20). Chou et al. (21) reported 67% MVI positive patients were found irregular boundary preoperatively. MVI was usually distributed on the edge of HCC (11). Previous studies (7, 9) also used these factors to predict MVI. Another predictive factor was tumor diameters. Almost all studies indicated that tumor diameter was associated with MVI. However, the classification of tumor diameter remained controversial. Kim et al. (22) suggested that tumor diameter more than 2 cm was risk factor of MVI. Siegel et al. (23) considered that tumor diameter more than 3 cm was risk factor of MVI. In our study, we made a more detailed division of tumor diameter by using optimal scale regression analysis in development group (10–35, 35–65, 65–120, 120–220 mm). Pawlik et al. found that the positive rate of MVI was 25, 40, 55, and 63% with tumor diameter less than 3 cm, between and 5 cm, between and 6.5 cm and more than 6.5 cm, respectively. This was basically consistent with our results.

In addition to the imaging analysis, we also evaluated the preoperative clinical factors. We found that ALP was associated with MVI. ALP was widely distributed in the liver. The increased value of ALP was associated with extrahepatic bile duct obstruction and intrahepatic space-occupying lesions. There were no studies reporting the correlation between ALP and MVI. A prospective study about the relationship between ALP and MVI was urgently needed.

Furthermore, we developed a user-friendly nomogram based on easy-accessible, non-invasive factors. Moreover, the nomogram showed satisfactory predictive performance of predicting MVI in both development group (C-index: 0.757) and validation group (C-index: 0.768) with favorable calibration. The use of our nomogram might be helpful to the surgeon in therapeutic decision making.

Our study had some limitations. First, it was a unicentric retrospective study and internal validation. Therefore, it should validate the results from the other centers. Second, the discomfort of the right upper abdomen should be classified in detail, but we didn’t find the related reports. Last, there were no quantitative prediction of MVI and no prediction of MVI classification.

## Conclusion

We developed and validated a nomogram for preoperative prediction of MVI. The nomogram incorporated clinical and imaging risk factors achieved favorable effectiveness in preoperatively predicting MVI of HCC patients.

## Data Availability Statement

The original contributions presented in the study are included in the article/**Supplementary Material**. Further inquiries can be directed to the corresponding authors.

## Ethics Statement

The study followed the Declaration of Helsinki. Because of the retrospective nature of the study, patient consent for inclusion was waived.

## Author Contributions

JRY, SZ, and JJY conceived and wrote the paper. LX, XQ, JWY, XH, YL, CW, and WGP collected and analyzed the data. LZ, MD, and WDP revised the whole paper. All authors contributed to the article and approved the submitted version.

## Funding

This work was supported by Sun Yat-Sen University Clinical Research Program (YHJH201910), China Postdoctoral Science Foundation (2020TQ0370), and National Science Foundation of China (grant no. 82002587).

## Conflict of Interest

The authors declare that the research was conducted in the absence of any commercial or financial relationships that could be construed as a potential conflict of interest.

## Supplementary Material

Supplementary Figure 1 Tumor size dividing by optimal scale regression analysis. According to the trend of the curve in the figure, tumor size was divided into 10–35, 35–65, 65–120, and 120–220 mm groups.

Supplementary Figure 2 The histopathological images of the microvascular invasion. The arrows point to the microvascular invasion. **(A)** ×100; **(B)** ×400.
