# Supplementary figures and images for: A Nomogram for Preoperative Estimation of Microvascular Invasion Risk in Hepatocellular Carcinoma: Single-Center Analyses With Internal Validation
Source: Front Oncol. 2021 Mar 4;11:616976. doi: 10.3389/fonc.2021.616976 (PMC7970183; doi:10.3389/fonc.2021.616976)

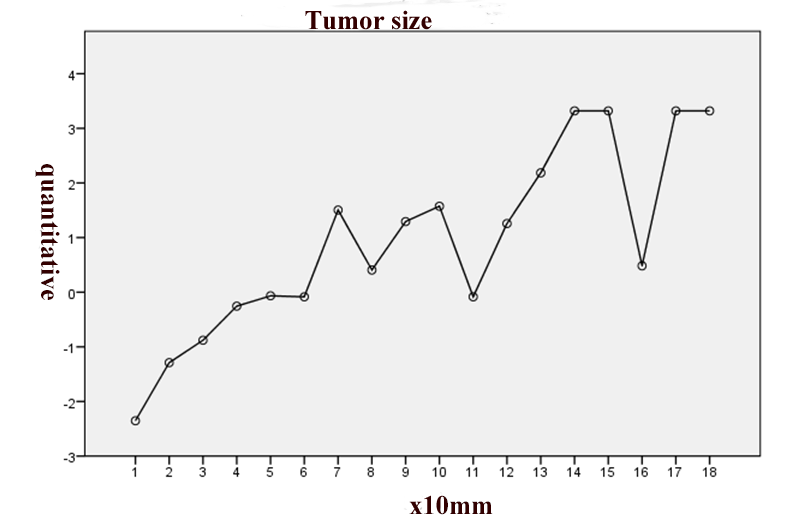

Supplement: Supplementary Figure 1 — Tumor size dividing by optimal scale regression analysis. According to the trend of the curve in the figure, tumor size was divided into 10–35, 35–65, 65–120, and 120–220 mm groups. [file Image_1.png]

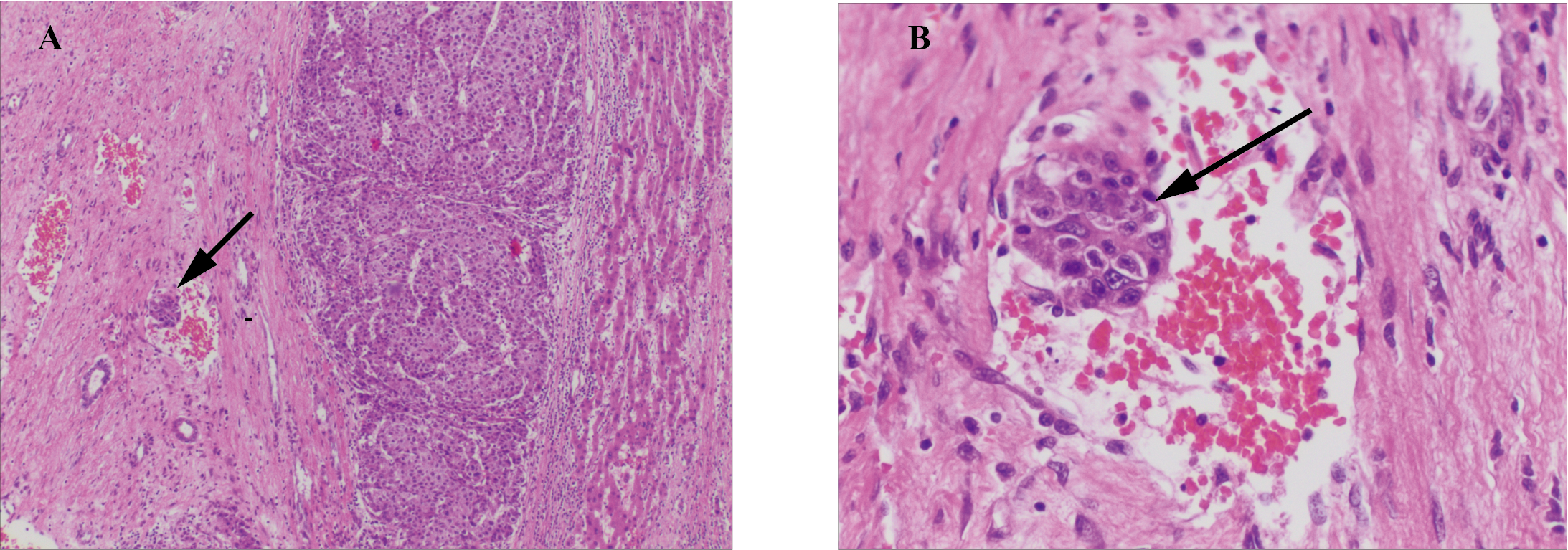

Supplement: Supplementary Figure 2 — The histopathological images of the microvascular invasion. The arrows point to the microvascular invasion. (A) ×100; (B) ×400. [file Image_2.png]
